# Helium-Induced Changes in Circulating Caveolin in Mice Suggest a Novel Mechanism of Cardiac Protection

**DOI:** 10.3390/ijms20112640

**Published:** 2019-05-29

**Authors:** Nina C. Weber, Jan M. Schilling, Moritz V. Warmbrunn, Mehul Dhanani, Raphaela Kerindongo, Jamila Siamwala, Young Song, Alice E. Zemljic-Harpf, McKenzie J. Fannon, Markus W. Hollmann, Benedikt Preckel, David M. Roth, Hemal H. Patel

**Affiliations:** 1 Department of Anesthesiology, Laboratory of Experimental Intensive Care and Anesthesiology (L.E.I.C.A.), Academic Medical Center, University of Amsterdam, Meibergdreef 9, 1105 AZ Amsterdam, The Netherlands; n.c.hauck@amsterdamumc.nl (N.C.W.); m.v.warmbrunn@amc.uva.nl (M.V.W.); r.p.kerindongo@amsterdamumc.nl (R.K.); m.w.hollmann@amsterdamumc.nl (M.W.H.); b.preckel@amsterdamumc.nl (B.P.); 2 VA San Diego Healthcare System and Department of Anesthesiology, University of California, San Diego, #125, 3350 La Jolla Village Dr., San Diego, CA 92161, USA; jan_schilling@gmx.net (J.M.S.); mdhanani@ucsd.edu (M.D.); jamila_siamwala@brown.edu (J.S.); NEARMYHEART@yuhs.ac (Y.S.); azemljicharpf@ucsd.edu (A.E.Z.-H.); mpavlich@ucsd.edu (M.J.F.); droth@ucsd.edu (D.M.R.); 3 Brown University and VA Providence, 830 Chalkstone Avenue, Providence, RI 02908, USA

**Keywords:** noble gas, helium, cardioprotection, caveolins, membranes, conditioning

## Abstract

The noble gas helium (He) induces cardioprotection in vivo through unknown molecular mechanisms. He can interact with and modify cellular membranes. Caveolae are cholesterol and sphingolipid-enriched invaginations of the plasma-membrane-containing caveolin (Cav) proteins that are critical in protection of the heart. Mice (C57BL/6J) inhaled either He gas or adjusted room air. Functional measurements were performed in the isolated Langendorff perfused heart at 24 h post He inhalation. Electron paramagnetic resonance spectrometry (EPR) of samples was carried out at 24 h post He inhalation. Immunoblotting was used to detect Cav-1/3 expression in whole-heart tissue, exosomes isolated from platelet free plasma (PFP) and membrane fractions. Additionally, transmission electron microscopy analysis of cardiac tissue and serum function and metabolomic analysis were performed. In contrast to cardioprotection observed in in vivo models, the isolated Langendorff perfused heart revealed no protection after He inhalation. However, levels of Cav-1/3 were reduced 24 h after He inhalation in whole-heart tissue, and Cav-3 was increased in exosomes from PFP. Addition of serum to muscle cells in culture or naïve ventricular tissue increased mitochondrial metabolism without increasing reactive oxygen species generation. Primary and lipid metabolites determined potential changes in ceramide by He exposure. In addition to direct effects on myocardium, He likely induces the release of secreted membrane factors enriched in caveolae. Our results suggest a critical role for such circulating factors in He-induced organ protection.

## 1. Introduction

Helium (He), a noble gas without anesthetic properties, induces profound protection against ischemia–reperfusion (I-R) injury in different organs of animals [1,2] and humans [3]. In both animals and humans, He preconditioning has shown to be cardioprotective in an early (directly to several minutes after He exposure) and late phase (20–24 h after He exposure) [3,4]. Although organ protection by He is observed in cell and animal models, and also in humans, data are sparse regarding a possible molecular mechanism of this cardiac protection. 

Previous work on ischemic and pharmacological preconditioning stimuli has shown that the sarcolemma localizes a variety of receptors whose stimulation leads to the triggering of cardiac protection. This trigger leads to activation of a number of mediators localized in the cytoplasm, such as tyrosine kinases (Src), prosurvival kinases (including phosphatidylinositol-3-kinase (PI3K)), protein kinase B (Akt) and glycogen synthase kinase 3b (GSK3b) [5]. Also, protein kinases such as protein kinase G (PKG), protein kinase A (PKA) and protein kinase C (PKC) isoforms, as well as nitric oxide synthase (NOS) and mitogen activated protein kinases (MAPK), have been shown to be crucially involved in preconditioning of the heart [5]. To date, it is accepted that all these mediators converge upon the mitochondria to modulate functions (including permeability transition pore (mPTP) function), energetics, reactive oxygen species (ROS) generation and ultimately provide cardiac protection [5].

Work from our group and others link He-induced cardiac protection to mitochondrial signaling pathways [6,7,8,9]. Moreover, emerging evidence shows that rapid coupling of the plasma membrane to mitochondria via caveolins simultaneously activates numerous parallel pathways important in cardiac protection [10,11,12]. Caveolae are cholesterol- and sphingolipid-enriched invaginations of the plasma membrane and are a subset of lipid rafts [13]. Caveolins, the structural proteins essential for caveolae formation, are present in three isoforms [14,15]: Caveolin-1 and -2 (Cav-1 and -2) are expressed in multiple cell types, while caveolin-3 (Cav-3) is found primarily in striated (skeletal and cardiac) muscle and certain smooth muscle cells [16]. Most importantly, caveolins have been shown to be critically involved in ischemic as well as volatile anesthetic-induced conditioning [17,18,19,20], and have been detected in mitochondria [10,21], where they modulate mitochondrial function [10,22,23]. It was previously shown that Cav-1 and Cav-3 levels are influenced by He pre and post conditioning in two different rat models [24,25]. Moreover, recently we were able to show that He preserves endothelial function by inducing the secretion of Cav-1 to the supernatant of human endothelial cells, with subsequent effects on endothelial permeability [26]. We hypothesized that the protective effect of helium is dependent on membrane modulation, and sought to determine the in vivo implication of He on the heart with a specific emphasis on membrane caveolin modulation in this study.

## 2. Results

### 2.1. Cardioprotection Ex Vivo

**Figure 1:**
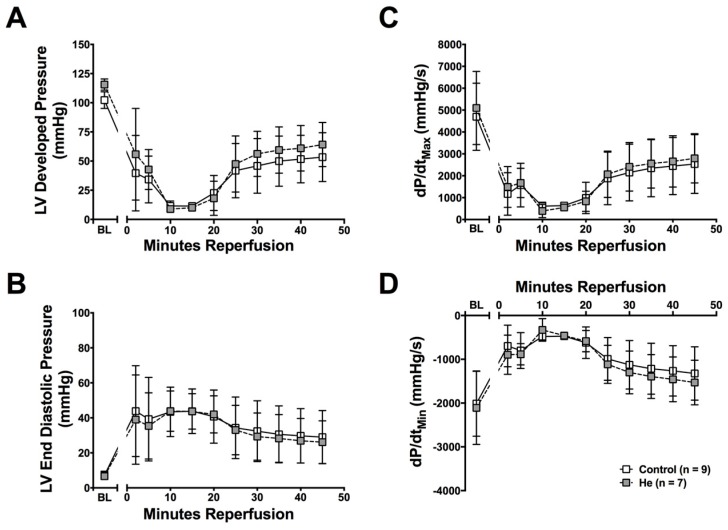
Langendorff perfused hearts. Helium did not result in increased recovery after global ischemia in the Langendorff perfused isolated heart 24 h after exposure. Cardiac function was measured as (**A**) left ventricular developed pressure (LVDP), (**B**) left ventricular end diastolic pressure (LVEDP), (**C**) contractility (dP/dt_max_) and (**D**) lusitropy (dP/dt_min_). Data are presented as mean ± standard deviation (SD) (*n* = 7 for helium, *n* = 9 for control) and results were considered significant when *p* < 0.05.

Numerous studies in vivo have shown a protective effect of He against cardiac ischemia–reperfusion injury [4,6,27], but no studies have explored if this is a direct effect on the heart. Cardiac function was evaluated 24 h after He or control (Ctrl) gas exposure using the Langendorff perfused heart. In this isolated heart preparation, all circulating factors and neuro-hormonal regulation of cardiac function are eliminated. Interestingly, no significant protective effect against ischemia-reperfusion injury was observed in the hearts from He-treated mice (Figure 1A–D) in this system. Therefore, He exposure does not result in cardiac protection from ischemia–reperfusion injury in the Langendorff perfused heart 24 h after exposure.

### 2.2. Circulating Protective Factors

**Figure 2:**
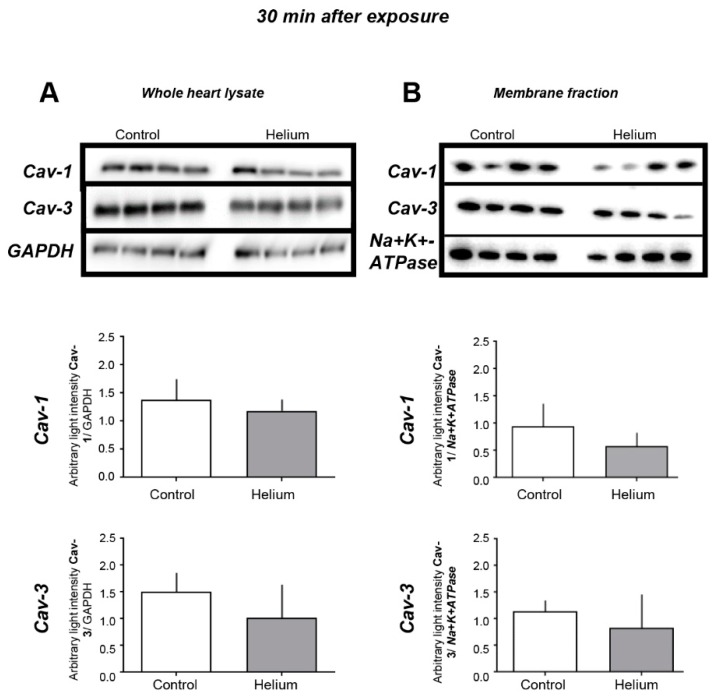
Cav-1 and Cav-3 levels in cardiac tissue 30 min post helium exposure. (**A**) Cav-1 and Cav-3 levels measured by western blot analysis in whole-heart tissue of mice as was the internal standard GAPDH, *n* = 8. (**B**) Cav-1 and Cav-3 levels measured by western blot analysis in membrane fractions of mice, as was the internal standard Na^+^K^+^-ATPase, *n* = 4. Data are shown as mean ± SD. No significant differences were observed in the different tissues 30 min post He exposure, suggesting that He does not have an effect on Cav-1 and Cav-3 levels in heart tissue 30 min post exposure.

**Figure 3:**
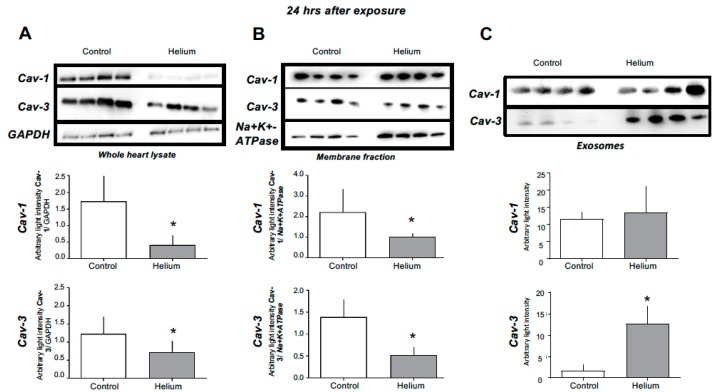
Cav-1 and Cav-3 levels in different tissues at the 24-h time point. (**A)** Cav-1 and Cav-3 levels were significantly lower in He whole-heart tissue of helium-treated mice, measured by western blot analysis. (**B**) Cav-1 and Cav-3 levels were significantly lower in membrane fractions of He-treated mice, measured by western blot analysis, *n* = 4. (**C**) Cav-3 levels were significantly higher in exosomes isolated from PFP of He-treated mice, measured by western blot analysis *n* = 4. Data are shown as mean ± SD, *p* < 0.05 was considered statistically significant. As Cav-1 and Cav-3 levels are lower in whole-heart lysate and membrane fractions but higher in exosomes of PFP at the same time point, these data suggest a secretion of Cav-1 and Cav-3 from cellular membranes located in the heart into the bloodstream.

Given the unexpected loss of He-induced protection in the isolated perfused heart, we hypothesized that He might induce protection through release of various circulating factors related to caveolin that have been shown to be protective in anaesthetic-induced cardiac protection [11,20,28]. We initially performed western blot analysis of the whole-heart tissue and membrane and cytosolic fractions of heart tissue, as well as exosome isolation from platelet free plasma (PFP). We observed no changes in Cav-1 or -3 in whole-heart lysates and membrane tissue fractions at 30 min post He exposure (Figure 2). However, when we assessed Cav-1 and Cav-3 protein levels in these compartments at 24 h post He inhalation, we observed a loss of Cav-1 and -3 in whole-heart lysates and membrane fractions in the He-exposed mice (Figure 3A,B, units are average light intensity), whole heart (Cav-1: 0.4 ± 0.3; Cav-3: 0.7 ± 0.3) compared with the time-matched control (Cav-1: 1.7 ± 0.7; Cav-3: 1.3 ± 0.4) (both *p* < 0.05), and membrane fraction (Cav-1: 1.0 ± 0.2; Cav-3: 0.5 ± 0.2) versus control (Cav-1: 2.2 ± 1.1; Cav-3: 1.4 ± 0.4) (both *p* < 0.05); no changes in cytosolic fraction were observed. Accordingly, exosomes isolated from PFP showed significantly increased Cav-3 expression and a tendency of increased Cav-1 in the He-treated mice (Figure 3C) (Cav-1: 13.5 ± 7.6; Cav-3: 12.7 ± 4.1, Cav-3 p<0.05) versus the time-matched control (Cav-1: 11.6 ± 2.1; Cav-3: 1.6 ± 1.4). These data suggest that He exposure produces caveolar-membrane-specific modifications leading to the release of circulating Cav-3-enriched factors that may potentiate the protective effects of He. 

### 2.3. Cav-1 and -3 Expression

**Figure 4:**
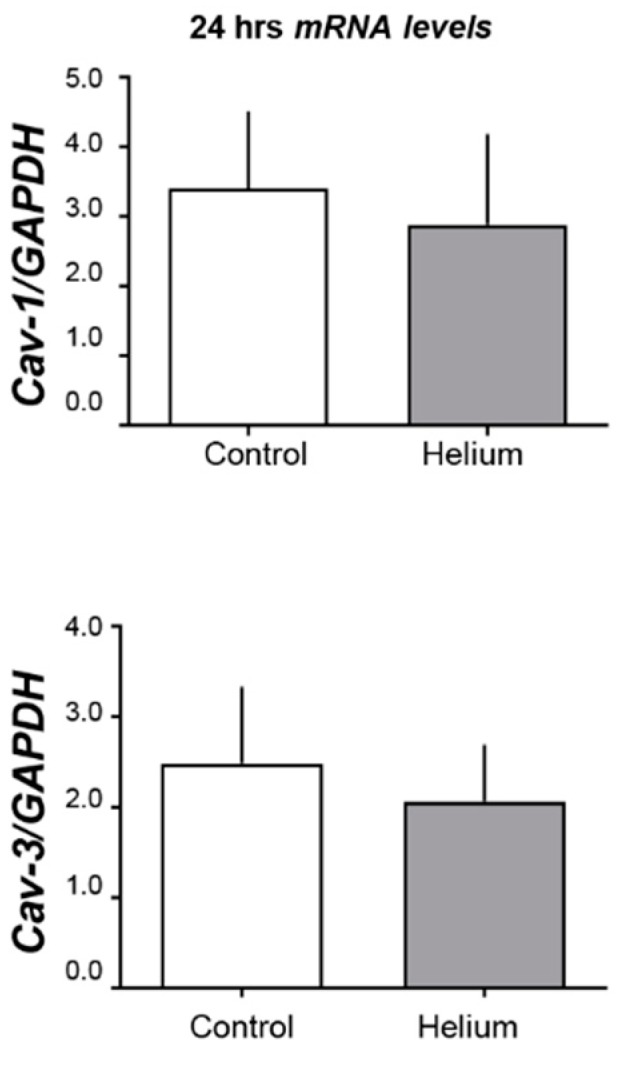
Cav-1 and Cav-3 mRNA levels. Histogram shows the results of the real-time PCR experiments of mice whole-heart tissue. Upper panel is Cav-1 over GAPDH. and lower panel is Cav-3 over GAPDH (*n* = 5 for control and *n* = 6 for helium). No significant changes were observed, suggesting that the increase of Cav-3 in exosomes from PFP at this time point is not due to higher levels of Cav-3 gene expression but through a different mechanism, such as the direct release of Cav-3 from plasma membranes.

Given the loss of caveolins from heart tissue and the increase in serum, we determined if the loss of caveolin in the heart was related to changes in mRNA. Cav-1 and -3 qRT-PCR was performed 24 h after He inhalation. Neither Cav-1 nor Cav-3 mRNA levels changed at this time point (Figure 4). These data suggest that the decreased levels of Cav-1 and -3 at the 24-h time point are caused rather by altered protein translocation/secretion than by transcriptional regulation.

### 2.4. Cardiac Ultrastructure, Cholesterol Quantity and Blood Plasma Fluidity

**Figure 5:**
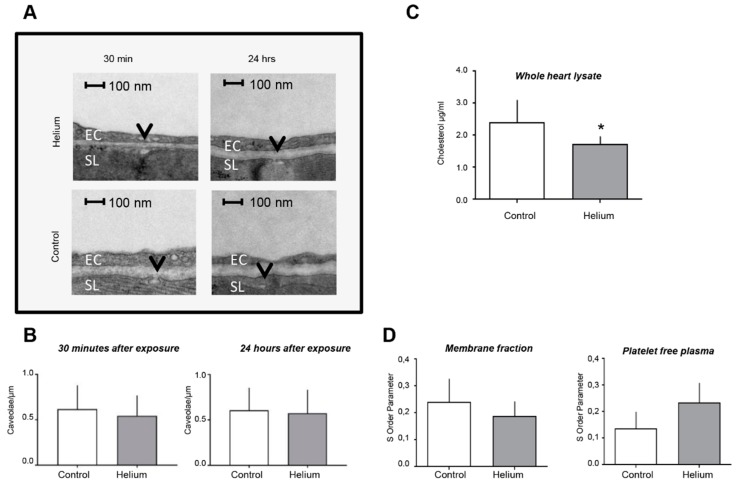
Assessment of cardiac ultrastructure and serum membrane fluidity post helium exposure. (**A**) Representative TEM images from left ventricular membranes of heart cells displayed per group, magnification 11,000 ×. Caveolae are indicated by the black arrow. EC: endothelial cell, SL: sarcolemma. No ultrastructural differences in cardiomyocytes were observed. (**B**) The number of caveolae in the sarcolemma of left ventricular myocytes are depicted as caveolae/µm for both time points, no significant differences were observed. All groups had the same sample size (*n* = 4 animals/group, *n* = 22–33 images acquired per animal). (**C**) Histogram shows the results of the cholesterol assay of whole-heart tissue in µM/mL. Twenty-four hours after helium exposure significantly less cholesterol was left in whole-heart tissue, *n* = 6 (control), *n* = 8 (helium). (**D**) The EPR histogram shows the changes in the S order parameter 24 h after He inhalation in membrane fractions and PFP, *n* = 4. The S order parameter is directly proportional to the membrane rigidity. No significant changes were observed. However, a trend towards decreased rigidity in the He group and increased rigidity in PFP can be appreciated when evaluating dedicated histograms. Data are shown as mean ± SD.

To further determine the impact of He exposure on cardiac ultrastructure, we performed transmission electron microscopy analysis of heart tissue and characterization of membrane fluidity and PFP. The analysis of images with 11,000× magnification on the left ventricle showed no differences in the number of caveolae/µm in sarcolemma (Figure 5A,B) at either 30 min or 24 h post He exposure. Further ultrastructural analysis focusing on the size, shape and organization of subsarcolemmal and intermyofibrillar mitochondria of cardiomyocytes showed no obvious changes. However, when the whole-heart lysate was assessed for cholesterol (a major structural component of membrane and caveolae), a significant decrease was observed 24 h after He inhalation (1.63 ± 0.05 vs. 2.39 ± 0.70 in controls; *p* < 0.05; Figure 5C) suggesting subtle changes to ultrastructure may be induced by He in the heart. 

Membrane fluidity was measured by electron paramagnetic spin resonance and a specific spin label. Membrane fractions of whole-heart tissue and PFP from the 24 h time point were prepared to determine membrane fluidity. 5-DSA was used as a spin label, and membrane fluidity was measured by the S-order parameter that is directly proportional to membrane rigidity. EPR spectra showed no significant changes after He administration. However, a trend towards decreased rigidity was observed in whole-heart tissue and a trend (*p* = 0.099) towards increased rigidity was measured in PFP (Figure 5D), again suggesting subtle alterations in membrane ultrastructure by He as a potential mechanism leading to protection dependent on circulating factors. 

### 2.5. Biological Effect of Helium-Conditioned Serum on Mitochondrial Function

**Figure 6:**
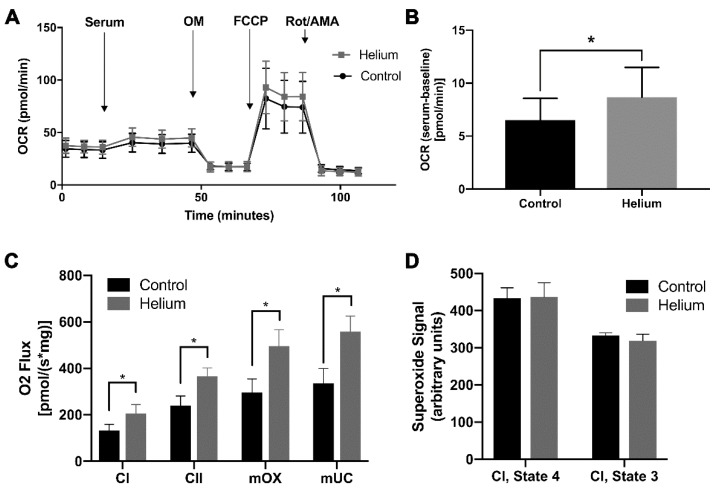
Biological effects of helium-conditioned serum on mitochondrial function. All assays utilized serum collected 24 h post exposure. (**A**) L6 muscle cells were treated with serum (5%) from control or helium-exposed mice. Seahorse Bioscience mitochondrial stress was assessed. Oxygen consumption rate (OCR) was measured before and after the addition of inhibitors (oligomycin, a complex V inhibitor; FCCP, a protonophore; and antimycin A and rotenone, complex III and I inhibitors, respectively) to derive parameters of mitochondrial respiration. (**B**) After addition of serum mean OCR was calculated after normalizing for baseline OCR of the L6 cells. (**C**) High-resolution respirometry analysis was performed using the Oroboros O2k-Respirometer. Serum from control and helium-exposed animals was incubated with naïve mouse heart ventricular tissue. Respiratory function of complex I and II activity (CI, CII), maximum oxidative phosphorylation capacity (mOX) and maximum uncoupled capacity (mUC) are shown after addition of complex I substrates glutamate and malate. (**D**) Electron paramagnetic resonance (EPR) with a DEPMPO spin probe was used to assess superoxide formation in isolated mouse heart mitochondria exposed to serum from control and helium-exposed animals under state 3 and 4 conditions with complex I substrates. All assays utilized *n* = 4–6 samples/group.

To determine if secreted factors in serum from helium-exposed mice have an impact on biological function, we specifically assessed the impact of serum on regulating mitochondrial function as mitochondrial function is critical in cardiac protection. We first assessed the impact of serum on the ability to alter mitochondrial metabolism of L6 muscle cells in culture using Seahorse metabolic flux assay. Addition of serum from helium-exposed mice resulted in a significant elevation of baseline metabolism in L6 muscle cells (Figure 6A,B). Given this change in mitochondrial respiration, we subsequently assessed the impact of serum on mitochondrial function on mouse ventricular tissue using the Oroboros O2k-Respirometer. Helium-conditioned serum resulted in increased mitochondrial respiration through complex I and II, maximum oxidative phosphorylation capacity, and maximum uncoupled capacity in isolated myocardial fibers in the presence of complex I substrates, glutamate and maleate (Figure 6C). To determine if this increased respiration was coupled and functional, we next assessed the impact of serum from control and helium-exposed animals on superoxide formation using electron paramagnetic resonance on isolated mouse cardiac mitochondria. We did not observe any significant difference in superoxide formation between control and helium-conditioned serum under state 3 or 4 conditions with complex I substrates (Figure 6D). These data suggest that helium-conditioned serum is able to functionally increase mitochondrial respiration. 

### 2.6. Primary and Complex Lipid Metabolites in Serum

**Figure 7:**
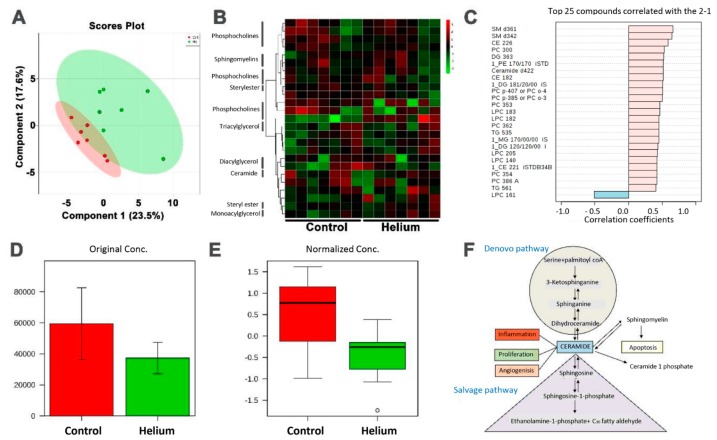
Complex lipid metabolites in serum. (**A**) Partial least squares discriminant analysis (PLS-DA) between He and control groups. (**B**) Heat map based on the normalized data and *p* values. (**C**) List of metabolites positively and negatively correlated with the He and control groups. (**D,E**) Removing the outliers determined by the Metaboanalyst outlier algorithm show the ceramide group to be differentially regulated in the He versus the control group. (**F**) Sphingolipid metabolic pathway associated with ceramide identified using pathway annotation.

No differences were observed in primary metabolites after correcting for false discovery rates early and late after He exposure (see supplementary metabolomics analysis report, Tables S2–S5, Figures S1 and S2). Next, we specifically assessed circulating lipid metabolites in He-treated versus the control group using liquid and gas chromatography. Evaluation of untargeted metabolomic profiling of He versus control group serum identified 174 known lipid metabolites (Table S2). The metabolites failed the FDR analysis with a lowest FDR value of 0.8, indicating no significant differences between the lipid metabolites of the control group and the helium group. There were principal component analysis similarities between the He and control groups. The partial least squares discriminant analysis (PLS-DA), which is a multivariate overfit model, also showed overlap between the He and control groups (Figure 7A). A heat map based on the normalized data and *p* values showed more green compared to red in the control group, indicating a lower abundance of some metabolites in the helium group compared with the control group (Figure 7B). Similarly, a list of metabolites positively and negatively correlated with the He and control groups is listed in Figure 7C. Removing the outliers as determined by the Metaboanalyst (Quebec, QC, Canada) outlier algorithm showed the ceramide group to be differentially regulated in He versus the control group (Figure 7D,E). The sphingolipid metabolic pathway is associated with ceramide, and may be important to lipid raft/caveolae regulation. This is represented by pathway annotation (Figure 7F).

## 3. Discussion

Helium has been shown to protect several organs in healthy and diseased models [1,2,29,30]. Previous in vivo studies already showed that three 5-min inhalations of 70% He and 30% oxygen prior to 30 min occlusion of the coronary artery and 3 h of reperfusion reduced cardiac infarct size in rabbits significantly [27]. Helium-induced cardioprotection was also observed in a rat in vivo study [4]. A recent study showed that seven days after combined He pre/post conditioning, Cav-3 levels in the rat heart decreased after an initial increase in a rat heart resuscitation model [24]. This might point to a translocation of Cav-3 in a time-dependent manner after He exposure. Changes in mRNA expression profiles after He post conditioning have been proposed to be one of the mechanisms underlying He-induced cardiac protection in ischemic models [25,31]. Proteins of the reperfusion injury salvage kinase (RISK) pathway have been implicated in He conditioning [25]. In the latter study, caveolin levels were also investigated in a He post conditioning ischemia–reperfusion model in a rat. Flick et al. showed that Cav-1 and Cav-3 levels increase after 15 min of helium post conditioning in the membrane fraction of myocardial tissue [25]. To our knowledge, this is the first study to suggest that helium-induced protection results in the release of circulating factors that are enriched in caveolins and are necessary to produce the protective phenotype. This is observed as a loss of this effect in the isolated heart preparation and the ability of the conditioned serum from helium-exposed animals to impact mitochondrial function in a number of preparations. 

### 3.1. Circulating Factors

In our study, we show that Cav-1 and Cav-3 levels are downregulated after 24 h in whole-heart tissue. Cav-1 is known to be expressed in many cell types, whereas Cav-3 is primarily present in striated muscle cells. Cav-1 has been linked to the signaling of ischemia–reperfusion damage, and Cav-3 is found to be critical for cardiac function [28]. With subfractionation of the whole heart, we determined that this loss was specific to the membrane fractions, with no change seen in cytosolic fractions. Corresponding to the decreased Cav-1 and Cav-3 levels in whole-heart tissue and in membrane fractions, we observed increased levels of Cav-3 in PFP and also of Cav-1, though not significant. Correlating with this, levels of cholesterol decreased in the membrane fractions 24 h after exposure, suggesting a secretion of caveolins and lipids from cardiac cell membranes into the blood stream. These findings are completely in line with our previous findings employing human endothelial cells to investigate the direct effects of He in vitro [26]. We showed that He in fact induces the release of caveolin to the supernatant of endothelial cells, and that the supernatant can preserve endothelial dysfunction by maintaining endothelial permeability [26]. Importantly, when we performed isolated heart studies in the present investigation, we demonstrated that the He-induced protection seen in the in vivo setting was no longer observed. Taken together these data, this strongly suggests that the loss of circulating factors may lead to a loss of protection in an isolated heart system. 

We used EPR to determine changes in membrane fluidity possibly caused by changes in structural membrane proteins. Our data show a trend towards decreased rigidity in whole-heart tissue from helium-treated mice 24 h after He inhalation, and a trend towards increased rigidity in PFP in the same group and time point. As caveolins are rigid structural membrane proteins, the trend towards a decrease of rigidity in He-treated whole-heart tissue, and the trend towards increased rigidity in PFP, support our hypothesis of secreted caveolins and lipids from tissue into the bloodstream. 

So far it has been challenging to find an agent that can effectively increase levels of caveolin to confer an organ-protective effect. Different models have already been used, such as directly targeting Cav-3 signaling with a Cav-3 scaffolding domain peptide or Cav-3 overexpression by an adenovirus, both which have led to cardioprotective effects [11,32]. Despite successful use of these techniques in preclinical models, such therapies may be difficult to translate into humans. 

### 3.2. Remote Protection 

As caveolins play a critical role in regulating cardiac-protective signals in plasma membrane, circulating caveolins could also interact with distant membrane proteins to yield cardiac protection. In the present study, we failed to show protection in the isolated perfused Langendorff heart after He inhalation, suggesting that the circulating component of He conditioning is critical to protection. In contrast, other groups have shown that injection of remote-conditioned serum from healthy volunteers induces protection ex vivo in the model of Langendorff [33,34]. Several mediators and targets involved in remote cardiac protection have been identified recently. Most prominently, cardioprotective factor X, microRNA, nitric oxide and exosomes have been discussed [35]. Interestingly, microvesicles and exosomes produced by different cell types via exocytosis contain apoptotic caspase 3, and may function as warning messengers for nearby cells in instances of reduced membrane integrity [36]. Moreover, the larger extracellular vesicles and endothelial-derived exosomes contain Cav-1, and the inhibition of caveolin-enriched microdomains blocks the release of exosomes [37]. Thus, caveolins could be the key component facilitating exosome secretion containing protective factors. We show that the serum from helium-conditioned mice has a biological benefit to enhance mitochondrial respiration without any significant increase in reactive oxygen species generation. Therefore, caveolins and the protective factors associated with caveolin in secreted particles may be essential to mediating remote protection.

Circulating caveolins could be of importance beyond the field of cardioprotection. Potential anticancer therapy, as chemotherapy shows increased levels of Cav-1 in cancer cells after chemotherapeutic exposure [38]. Most importantly, in certain cancer types it has been shown that caveolin secreted by prostate cancer cells might stimulate cell survival and angiogenic activities [39,40,41,42]. These studies thereby suggest a role for secreted caveolin as a mediator of cell survival [41]. Elevation or knockdown of Cav-1 expression plays a critical role in chemosensitivity [43]. Because Cav-1 is upregulated during cell stress, it has been proposed that Cav-1 is a stress-related molecule that can be upregulated by various mechanisms [44]. It is also possible that Cav-1 may induce such effects by regulating interaction with other cell proteins (e.g., calpain) to modulate the stress responses that control cellular process, such as autophagy and mitochondrial protection, which may be critical to acute adaptation from ischemia–reperfusion injury [45]. 

As circulating factors other than caveolins are proposed to play a role in cardiac conditioning in blood [33], consideration of other protective mediators is also necessary. Therefore, we also analyzed primary metabolites and complex lipid metabolites from He-conditioned serum to identify potential changes in metabolic patterns. We did not observe any significant changes in early (30 min) or late (24 h) primary metabolites post He exposure, suggesting the likelihood that other blood components (i.e., exosomes, bioactive lipids, proteins, etc.) that we did not analyze could be the missing factors for the observed protective effects of He. The altered EPR signal in the PFP samples (which are enriched in microparticles, exosomes, etc.) suggest altered lipid content, and future studies should focus on the biological function of cell-derived membranes in these compartments.

Ceramide synthases play a critical role in sphingolipid metabolism and the formation of endocytic vesicles. The gene responsible for the expression of ceramide synthases is the longevity assurance gene (LAG1), identified in a yeast-aging model. Deletion of the LAG1 gene results in the extension of lifespan in yeast [46]. Ceramides added to ATP-depleted macrophages result in the formation of endocytic vesicles that are not caveolin positive [47]. When caveolins are synthesized in the endocytic vesicles, they form caveolae. Overall, He induction increases caveolae formation, and the release may involve ceramides and the sphingolipid metabolism. How caveolins are added to the endocytic vesicle and transported outside cells into the blood stream needs further investigation. 

In the present study, we aimed to contribute to the understanding of the mechanism behind He-induced cardiac protection. One major limitation in this study was that the animals used were relatively young and healthy. If the proposed mechanism is to be translated to patients suffering from heart disease or at risk of heart disease, similar conditions should be mimicked in the animal experiment. As our findings are limited to one species of healthy mice, further confirmation of our findings should be explored in cells and aged animal models. Additionally, we used Na^+^/K^+^ATPase as a housekeeping protein and normalized Cav-1 and Cav-3 to this. It is possible that longer time points post helium exposure have more profound effects on membrane ultrastructure impacting normalization controls. This is an area that has not been well investigated in the literature and may warrant focus in future studies.

## 4. Materials and Methods 

### 4.1. Animal Handling

All animal experiments were approved by the Institutional Animal Care and Use Committee (IACUC) at the VA San Diego Healthcare System. Animals were treated in accordance with the Guide for the Care and Use of Laboratory Animals (NIH Publication No. 85-23, revised 1996). Furthermore, all experiments where performed according to the ARRIVE guidelines. C57BL/6J mice were purchased from Jackson Laboratory (Bar Harbor, ME, USA) at eight weeks of age, and all experiments were performed in young male mice between 8–12 weeks of age. Animals were kept on a 12-h light/dark cycle in a temperature- and humidity-controlled room with ad libitum access to food and water.

### 4.2. General Chemicals and Solutions

If not otherwise stated, all chemicals and solutions were purchased from Sigma-Aldrich Corp. (St. Louis, MO, USA), Santa Cruz Biotechnology (Dallas, Texas, USA), BD Bioscience (San Jose, CA, USA) or Millipore (Massachusetts, MA, USA).

### 4.3. Anesthesia Exposure Protocol and Tissue Collection

**Figure 8:**
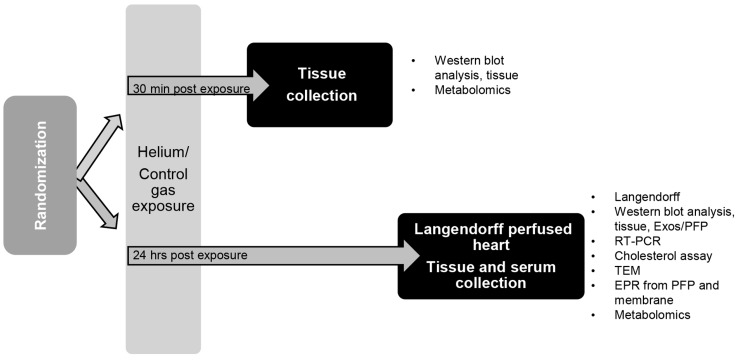
Experimental protocol overview of organ and blood collection. C57BL/6J mice were randomized into two different groups. Subsequently, mice inhaled either 70% He or 30% oxygen for 30 min in a Plexiglas chamber. Thirty minutes or 24 h post exposure, blood was drawn and hearts were excised for further analysis. PFP: platelet free plasma, PE: post exposure.

Animals were exposed in a standard cage plexiglass chamber (#7060R, Columbus Instruments, OH, USA) for 30 min at a flow of 5 L/min to either a treatment of 70% He/30% oxygen mix, or a concentration of 30% oxygen in air as a control group. After 30 min or 24 h, blood was collected through cardiac puncture and hearts were excised under deep pentobarbital (50 mg/kg i.p.) anaesthesia, flash frozen in liquid nitrogen and stored at –80 °C until further processing (Figure 8). 

### 4.4. Langendorff Perfused Heart Model

Twenty-four h after He or Ctrl exposure, mice were anesthetized with sodium pentobarbital (50 mg/kg i.p.), the heart excised and the aorta cannulated for Langendorff perfusion of the coronary circulation, as described previously [48,49]. All hearts were perfused at a hydrostatic pressure of 80 mmHg with modified Krebs–Henseleit buffer bubbled with 95% O_2_/5% CO_2_ at 37 °C, containing: 119 mM NaCl, 22 mM NaHCO_3_, 4.7 mM KCl, 2.5 mM CaCl_2_, 1.2 mM MgCl_2_, 1.2 mM KH_2_PO_4_, 11 mM D-glucose and 0.5 mM EDTA. For the ischemia–reperfusion (I-R) protocol, a 20-min normoxic stabilization at intrinsic heart rates was followed by ventricular pacing at 7 Hz. After an additional 10 min, baseline measurements were made before subjecting hearts to 25 min of global, normothermic ischemia, followed by 45 min aerobic reperfusion with pacing re-introduced after 2 min.

### 4.5. Immunoblot Analysis 

Immunoblot analyses were performed as previously described [50]. Flash-frozen tissue was homogenized on dry ice using a metallic potter. After re-suspending the tissue powder in lysis buffer containing 10 mM Hepes, 2 mM EDTA and 1 mM MgCl_2_, the solution was passed through a 21-gauge needle and subsequently sonicated at 30% amplitude on ice three times for 10 s. Protein concentration was determined by Bradford analysis (BioRad; Hercules, CA, USA) using spectrophotometry at a wavelength of 595 nm on an Infinite M200 plate reader (Tecan; San Jose, CA, USA). Protein was separated on a 10%–12% polyacrylamide precast gel. Proteins were transferred to a polyvinylidene difluoride membrane by electroblotting. Membranes were blocked in tris-buffered saline with 1% tween (TBST) containing 3% bovine serum albumin (BSA) solution. Afterwards, the membrane was incubated with the respective primary antibody in 3% BSA (1:1000, mouse antibody anti-Cav-3, mouse anti-Cav-1 and rabbit anti-GAPDH 1:5000) at 4 °C overnight. Primary antibodies were visualized using secondary antibodies (1:5000 in TBST) conjugated with horseradish peroxidase from Santa Cruz Biotechnology. The enhanced chemiluminescent reagent from Amersham Biosciences (Piscataway, NJ, USA) was used for the detection of protein bands. In comparison to a molecular weight standard (Santa Cruz Biotechnology, Dallas, TX, USA), the appropriate sizes of the bands were proven.

### 4.6. Cholesterol Assay

The cholesterol assay of whole-heart tissue was performed using the Amplex Red Cholesterol assay kit from Invitrogen (Carlsbad, CA, USA) according to the manufacturer’s instructions. After 60 min incubation (37 °C, in the dark), fluorescence was measured. Cholesterol levels were determined from cholesterol standard curves.

### 4.7. Quantitative Real-Time Polymerase Chain Reaction (RT-PCR)

About 30 mg of heart tissue was flash frozen in liquid nitrogen then pulverized with a cold mortar. From the 24-h time point, hearts were used for the RT-PCR analysis. Subsequently, the powder was homogenized in Tripure isolation reagent (Roche, the Netherlands). Total RNA was extracted from 100 µl homogenate using the NucleoSpin RNA Midi kit (Macherey-Nagel, The Netherlands) according to the manufacturer’s protocol. Next, RNA concentration was determined by UV-Vis absorbance spectroscopy NanoDrop 2000 (Thermo Scientific, Rockford, IL, USA). According to manufacturer’s instructions, 300 ng of RNA was reverse transcribed to cDNA using the Transcriptor First Strand cDNA Synthesis Kit, and 1 µl cDNA was used in a total volume of 10 µL PCR mix per reaction. Each mixture contained 10 µM of primer pairs (Table S1) and 2× LightCycler 480 SYBR Green I Master (Roche). Real-time qPCR amplification was carried out using the LightCycler 480 instrument (Roche) under the following conditions: pre-incubation at 95 °C for 10 min, followed by 42 cycles of 95 °C for 15 s, 56 °C for 10 s and 72 °C for 15 s. 

Analysis of raw data was conducted using the programs LC480Converter and LinRegPCR. For each sample, PCR efficiency was determined. Subsequently the means of the PCR efficiencies per target were used to calculate the estimated starting concentration per sample. Afterwards, each target gene was normalized to the housekeeping gene GAPDH [51]. 

### 4.8. Isolation of Membrane and Cytosolic Fractions 

Flash-frozen tissue was homogenized as described in Section 4.5. After sonication, the suspension was centrifuged at low speed (500 *g*) for 10 min at 4 °C to pellet cell debris. The supernatant was centrifuged in an Optima Max tabletop ultracentrifuge (Beckmann Coulter Inc., Brea, CA, USA, swinging rotor TLS 55) for 30 min at 37,000 *g*. The resulting pellet was re-suspended in hypotonic lysis buffer and used as the particulate fraction for immunoblotting and EPR. The supernatant was again centrifuged at the same speed for 30 min and the resulting supernatant was used as cytosolic fraction for immunoblotting. 

### 4.9. Isolation of Serum, Platelet Free Plasma and Exosomes 

Serum was collected as described in Section 4.3 and subsequently isolated to clot at room temperature for 30 min in Becton Dickinson serum collection tubes. After this, blood was centrifuged at 1200 *g* for 15 min at room temperature. The supernatant was used as serum. 

PFP blood was collected in citrate buffer and centrifuged at 1200 *g* for 15 min at room temperature. The supernatant was collected, and a further centrifugation step was performed at 12,000 g for 12 min. The supernatant, platelet free plasma (PFP), was further analyzed using electron paramagnetic resonance spectrometry. Furthermore, exosomes were isolated from PFP. PFP was centrifuged at 20,000 *g* at 4 °C for 40 min in the ultra mini centrifuge (Optima Max tabletop centrifuge TLA 55 rotor SN12/1150) using no brake.

The supernatant was again centrifuged at 100,000 *g* at 4 °C with no brake for 95 min.

The pellet containing exosomes was directly suspended in high pH buffer (150 mM Na_2_CO_3_ and 1 mM Na EDTA). Each sample was than sonicated on ice for 3 × 15 s at 30% power and further processed for standard western procedure.

### 4.10. Electron Paramagnetic Resonance Spectrometry (EPR) 

As previously described, hydrocarbon chain mobility was measured using fatty acid spin labelling EPR analysis with 5-DOXYL-stearic acid (5-DSA) as a spin probe [52,53,54]. The number designation indicates the relative position of the nitroxide on the stearic acid relative to the polar carboxylic group. 5-DSA probes membrane rigidity close to the hydrophilic surface of the membrane [55]. Platelet free plasma or crude membrane extracts of the heart tissue were incubated for 10 min with a specific spin label (1 mM final concentration) at room temperature (25 °C). The mixture was then loaded into a 50-µL glass capillary tube and inserted into the EPR cavity of a MiniScope MS300 Benchtop spectrometer (Magnettech, Berlin, Germany). The MiniScope was maintained at 25 °C, and the EPR spectra were measured. EPR conditions were the following: microwave power, 5 mW; modulation amplitude, 2 G; modulation frequency, 100 kHz; sweep width, 150 G centred at 3349.0 G; scan rate, 7.5 G/s, with each spectrum representing the average of 20 scans. The fluidity parameters T∥ and T⊥ were used to calculate the order parameter as previously described [54,55].

For superoxide measurement studies, mitochondria were isolated as previously published [10]. Isolated mitochondria (0.1–0.2 mg of protein) were incubated with the control or helium serum (5%) for 5 min, then with 70 mM 5-(diisopropoxyphosphoryl)-5-ethyl-1-pyrroline-N-oxide (DEPMPO, 5 min). After addition of the appropriate combinations of substrates, the mixture was loaded into 500-µl glass capillary tubes and introduced into the EPR cavity of a MiniScope MS300 benchtop spectrometer (Magnettech GmbH, Berlin, Germany). We confirmed that the detected EPR signals were substrate specific, and not due to redox cycling in the studied mixtures, by noting a lack of signals when DEPMPO was mixed with combinations of substrates and inhibitors in the absence of mitochondria. Assignment of the observed signals from mitochondria was confirmed through computer-assisted spectral simulation using the WinSim software (http://epr.niehs.nih.gov/pest.html). Validation of the signal was performed as previously described [10]. Signals were quantified by measuring the peak amplitudes of the observed spectra.

### 4.11. Transmission Electron Microscopy (TEM)

Cardiac tissue was assessed via TEM in four hearts of each group, with specific emphasis on the quantification of caveolae on the sarcolemma and mitochondrial morphology. Animals were anesthetized with pentobarbital (50 mg/kg i.p.) and transcardial perfusions (5 mL/min) were performed in deeply anesthetized animals initially with 37 °C perfusion buffer (3 min, Ca2^+^ and Mg2^+^ free DPBS, 10 mM HEPES, 0.2 mM EGTA, 0.2% BSA, 5 mM glucose and 9.46 mM KCl), followed by 37 °C fixative (3 min freshly prepared 2.5% glutaraldehyde, 2% paraformaldehyde in 0.15 M cacodylate buffer) using a peristaltic pump (Langer Instruments, Boonton, NJ, USA) as described previously [56]. The left ventricular free wall was dissected immediately, and small tissue pieces were immersed in fixative for 2 h at room temperature, followed by overnight incubation in fixative at 4 °C. Samples were post-fixed in 1% OsO_4_ in 0.1 M cacodylate buffer, enbloc stained with uranyl acetate (2%−3%), dehydrated in ethanol, embedded in longitudinal orientation in Durcupan epoxy resin (Sigma-Aldrich) and polymerized for 48 h at 60°. Blocks were sectioned at 50–60 nm on a Leica UCT ultramicrotome (Wetzlar, Germany), picked up on Formvar and carbon-coated copper grids and stained in uranyl acetate and lead citrate prior to TEM photography (Jeol 1200 EX-II; Jeol Ltd., Akishima, Japan). Blinded observers acquired scans of four capillaries with surrounding sarcolemma per animal (*n* = 4 hearts per group, total capillaries *n* = 24) at 1900×, 2900× and 11000× magnifications using a Tecnai G2 Spirit BioTWIN (FEI, Hillsboro, OR, USA) transmission electron microscope. Images were captured using an Eagle 4k HS digital camera (FEI). The total density of caveolae/100 µm cardiomyocyte sarcolemma was quantified using ImageJ (National Institutes of Health, Bethesda, MD, USA). 

### 4.12. Seahorse Mitochondrial Respiration Assay 

The ability of serum to alter cellular metabolism was performed as previously published [57]. Briefly, oxygen consumption rates (OCR) from a monolayer of adherent L6 muscle cells purchased from ATCC Cat. # CRL-1458), were measured using a Seahorse XF96 analyzer. The L6 cells were seeded at 20,000 cells/well (passage 3−4) on a 96-well tissue culture plate (Seahorse Biosciences, North Billerica, MA, USA) 24 h before the OCR measurements. The bioenergetic profile was derived by acutely injecting control or helium serum (5%) through the first of the four injection ports, followed by the modulators of the electron transport chain. The OCR was calculated by plotting the O_2_ tension of the media as a function of time (pmol/min).

### 4.13. High-Resolution Mitochondrial Respirometry

Mitochondrial function in manually dissected fibers from the left ventricular free wall of mouse hearts were evaluated using an Oroboros O2k-Respirometer. Heart samples weighed between 0.5 and 1.0 mg. Serum (5%) from the control and helium-exposed animals was incubated in the oximetry chamber along with ventricular tissue in media containing: 100 mM KCl, 75 mM mannitol, 25 mM sucrose, 5 mM H_3_PO_4_, 0.05 mM EDTA and 10 mM Tris HCl; pH 7.2, at 37 °C. After 10 min of equilibration, respiratory function of complex I and II activity (CI, CII), maximum oxidative phosphorylation capacity (mOX) and maximum uncoupled capacity (mUC) were determined after addition of complex I substrates, glutamate and malate. Oxygen use trace and rate determinations were obtained using DatLab 7 software and normalized to protein.

### 4.14. Metabolomics

Serum metabolomic analysis was performed by the West Coast Metabolomics Center, UC Davis Genome Center, University of California Davis, 451 Health Sciences Drive, Davis, California 95616, United States. An unbiased assessment of primary and complex lipid metabolites were identified in serum as per center protocols. Data were subsequently analyzed with MetaboAnalyst software 3.0.

### 4.15. Statistical Analysis

All data are Mean ± SD. Statistical analysis was performed in GraphPad Prism (GraphPad Software version 5.01, La Jolla, CA, USA) using one-way ANOVA and two-way ANOVA with Bonferroni post hoc tests for multiple comparisons. Data were tested for normality distribution using D’Agostino and Pearson normality testing. Normally distributed data of two groups were compared using an unpaired t-test (with Welch’s correction) and not normally distributed data were compared using a Mann–Whitney test. *p* < 0.05 was considered significant. 

## 5. Conclusions

The data of the present study show that after He inhalation, Cav-1 and -3 circulate in the blood, and that no cardiac protection could be achieved in isolated hearts without these circulating blood components. The current data add mechanistic insight to recently published data showing that He can affect caveolin release in human cells. The current work suggests a critical role for circulating factors in He-induced organ protection. 

## Supplementary Materials

Supplementary materials can be found at https://www.mdpi.com/1422-0067/20/11/2640/s1.

## Abbreviations

Cav-1	Caveolin-1
Cav-2	Caveolin-2
Cav-3	Caveolin-3
He	Helium
h	Hours
min	Minutes
Ctrl	Control
PFP	Platelet free plasma
RISK	Reperfusion Injury Salvage Kinase
