# Supplementary material for: Helium-Induced Changes in Circulating Caveolin in Mice Suggest a Novel Mechanism of Cardiac Protection
Source: Int J Mol Sci. 2019 May 29;20(11):2640. doi: 10.3390/ijms20112640 (PMC6600664; doi:10.3390/ijms20112640)
Supplement: Supplementary file 1 [file ijms-20-02640-s001.pdf]

Table S1. Used primers: according to FASEB J. 2007 May;21(7):1565-74.

Caveolin-1	
Forward	ACGACGACGTGGTCAAGATT
Reverse	GTGCAGGAAGGAGAGAATGG
Caveolin-3	
Forward	TCACCACGTTCACCGTCTCC
Reverse	GTGCAGGAAGGAGAGAATGG
GAPDH	
Forward	TGAAGCAGGCATCTGAGGG
Reverse	CGAAGGTGGAAGAGTGGGAG

Table S2. Circulating lipid metabolites were profiled in He treated vs control group using liquid and gas chromatography.

PubChem_ID	Compound_Name	Family	Fold change (He/WT)	Correlation	*t*-stat	*p*-value	FDR
5348046	1_CE (22:1)	Cholesteryl ester	1.0	0.6	2.9	0.01	0.8
0	1_Ceramide C17	Ceramide	1.0	0.6	2.5	0.03	0.8
4458433	1_Cholesterol d7	Cholesterol	1.0	0.5	2.1	0.06	0.8
5 5314029	1_CUDA (pos)	N-cyclohexyl-N′-dodecanoic acid urea	1.0	0.5	2.1	0.06	0.8
2297877	1_DG (12:0/12:0/0:0)	Diacyglycerol	1.0	0.5	2.1	0.06	0.8
4 151838	1_LPC (17:0)	Monoacylglycerophosphocholines	1.0	0.5	2.0	0.07	0.8
2477946 3	1_LPE (17:1)	Monoacylglycerophosphoethanolamines	1.0	0.5	1.8	0.09	0.8
4260746	1_MG (17:0/0:0/0:0)	Monoacyglycerol	1.0	0.5	1.8	0.10	0.8
4 107036	1_PC (25:0)	Diacylglycerophosphocholines	1.0	0.5	1.8	0.11	0.8
2477851 6	1_SM (d35:1)	Ceramide phosphocholines (sphingomyelins)	1.0	0.4	1.7	0.12	0.8
4689176 3	1_TG (17:0/17:1/17:0)-d5	Triacylglycerols	1.0	0.4	1.6	0.13	0.8
5283479	Acylcarnitine C18:1	Acylcarnitine	1.0	0.4	1.6	0.13	0.8
5347783	CE (16:1)	Steryl esters	0.7	0.4	1.6	0.15	0.8
0	Ceramide (d42:2)	Ceramide	0.7	0.4	1.4	0.19	0.8
2283354	Cholesterol	Cholesterol	1.0	0.4	1.4	0.19	0.8
3	DG (36:2)	Diacyglycerol	0.8	0.4	1.4	0.20	0.8
5283568 5997	GlcCer (d40:1)	Simple Glc series (Sphingolipids)	0.9	0.4	1.4	0.20	0.8

9543722 6321360	LPC (15:0)	Monoacylglycerophosphocholines	1.0	0.4	1.3	0.22	0.8
460602 452110	PC (16:0/9:0(CHO))	Diacylglycerophosphocholines	0.6	0.3	1.0	0.32	0.8
5292489 3	PE (34:2)	Diacylglycerophosphoethanolamines	0.9	0.2	0.7	0.49	0.9
9939941 9543993	SM (d33:1)	Ceramide phosphocholines (sphingomyelins)	0.9	0.2	0.6	0.55	0.9
2524046	TG (49:1)	Triacylglycerols	1.0	0.1	0.4	0.70	1.0
0	TG (49:2)	Triacylglycerols	1.1	0.1	0.4	0.73	1.0
9544045	TG (49:2)	Triacylglycerols	0.8	0.1	0.4	0.73	1.0

Supplementary File: Analysis report metabolomics 30 min

Metabolomic Data Analysis with MetaboAnalyst 3.0

1. Background

The Pathway Analysis module combines results from powerful pathway enrichment analysis with the pathway topology analysis to help researchers identify the most relevant pathways involved in the con- ditions under study.

There are many commercial pathway analysis software tools, such as Pathway Studio, MetaCore, or Ingenuity Pathway Analysis (IPA), etc. Compared to them, the pathway analysis module was specially developed for metabolomics studies. It uses the high-quality KEGG metabolic pathways as the backend knowledgebase. It integrates many well-established (i.e. univariate analysis, over-representation analysis) methods, as well as novel algorithms and concepts (i.e. Global Test, GlobalAncova, network topology analysis) into pathway analysis. Another feature is a Google-Map style interactive visualization system to deliver the analysis results in an intuitive manner.

2. Data Input

Pathway Analysis accepts either a list of compound labels (common names, HMDB IDs or KEGG IDs) with one compound per row, or a compound concentration table with samples in rows and compounds in columns. The second column must be phenotype labels (binary, multi-group, or continuous). The table is uploaded as comma separated values (.csv).

3. Compound Name Matching

The first step is to standardize the compound labels used in user uploaded data. This is a necessary step since these compounds will be subsequently compared with compounds contained in the pathway library. There are three outcomes from the step - exact match, approximate match (for common names only), and no match. Users should click the textbfView button from the approximate matched results to manually select the correct one. Compounds without match will be excluded from the subsequently pathway analysis.

Table S3. shows the conversion results. Note: *1* indicates exact match, *2* indicates approximate match, and *0* indicates no match. A text file contain the result can be found the downloaded file *name map.csv*.

Table S3. Result from Compound Name Mapping (both time points).

	Query	Match	HMDB	PubChem	KEGG	Comment
1	C00312	L-Threo-2-pentulose	HMDB00751	22253	C00312	1
2	C00181	D-Xylose	HMDB00098	135191	C00181	1
3	C00379	D-Xylitol	HMDB02917	6912	C00379	1
4	C00385	Xanthine	HMDB00292	1188	C00385	1
5	C00183	L-Valine	HMDB00883	6287	C00183	1
6	C00299	Uridine	HMDB00296	6029	C00299	1
7	C00366	Uric acid	HMDB00289	1175	C00366	1
8	C00086	Urea	HMDB00294	1176	C00086	1
9	C00106	Uracil	HMDB00300	1174	C00106	1
10	C00043	Uridine diphosphate-N-	HMDB00290	445675	C00043	1
11	C00082	L-Tyrosine	HMDB00158	6057	C00082	1
12	C00078	L-Tryptophan	HMDB00929	6305	C00078	1
13	C01157	4-Hydroxyproline	HMDB00725	5810	C01157	1
14	C00376	Retinal	HMDB01358	638015	C00376	1
15	C00178	Thymine	HMDB00262	1135	C00178	1
16	C00214	Thymidine	HMDB00273	5789	C00214	1
17	C00188	L-Threonine	HMDB00167	6288	C00188	1
18	C01620	Threonic acid	HMDB00943	151152	C01620	1
19	C00245	Taurine	HMDB00251	1123	C00245	1
20	C00059	Sulfate	HMDB01448	1117	C00059	1
21	C00089	Sucrose	HMDB00258	5988	C00089	1
22	C00042	Succinic acid	HMDB00254	1110	C00042	1
23	C01530	Stearic acid	HMDB00827	5281	C01530	1
24	C00794	Sorbitol	HMDB00247	5780	C00794	1
25	C00065	L-Serine	HMDB00187	5951	C00065	1
26	C00818	NA	NA	NA	NA	0
27	C01685	Ribonic acid	HMDB00867	5460677	C01685	1
28	C00022	Pyruvic acid	HMDB00243	1060	C00022	1
29	C00013	Pyrophosphate	HMDB00250	644102	C00013	1
30	C02067	Pseudouridine	HMDB00767	15047	C02067	1
31	C00148	L-Proline	HMDB00162	145742	C00148	1
32	C03844	D-Pinitol	HMDB34219		C03844	1
33	C02656	Pimelic acid	HMDB00857	385	C02656	1
34	C00346	O-Phosphoethanolamine	HMDB00224	1015	C00346	1
35	C00009	NA	NA	NA	NA	0
36	C05332	Phenylethylamine	HMDB12275	1001	C05332	1
37	C00079	L-Phenylalanine	HMDB00159	6140	C00079	1
38	C01601	Pelargonic acid	HMDB00847	8158	C01601	1
39	C00802	Oxalureate	METPA0081		C00802	1
40	C08362	Palmitoleic acid	HMDB03229	445638	C08362	1
41	C00249	Palmitic acid	HMDB00220	985	C00249	1
42	C01879	Pyroglutamic acid	HMDB00267	7405	C01879	1
43	C00209	Oxalic acid	HMDB02329	971	C00209	1
44	C00077	Ornithine	HMDB00214	6262	C00077	1
45	C00712	NA	NA	NA	NA	0
46	C02721	NA	NA	NA	NA	0
47	C00153	Niacinamide	HMDB01406	936	C00153	1
48	C00645	N-Acetylmannosamine	HMDB01129	11096158	C00645	1
49	C03878	Beta-N-Acetylglucosamine	HMDB00803	24139	C03878	1
50	C06424	Myristic acid	HMDB00806	11005	C06424	1
51	C00137	Myoinositol	HMDB00211		C00137	1
52	C00159	D-Mannose	HMDB00169	18950	C00159	1
53	C00711	NA	NA	NA	NA	0
54	C07272	NA	NA	NA	NA	0
55	C00476	D-Lyxose	METPA0046		C00476	1
56	C00532	L-Arabitol	HMDB01851	439255	C00532	1
57	C00047	L-Lysine	HMDB00182	5962	C00047	1
58	C01595	Linoleic acid	HMDB00673	5280450	C01595	1
59	C00123	L-Leucine	HMDB00687	6106	C00123	1
60	C02679	Dodecanoic acid	HMDB00638	3893	C02679	1
61	C01432	NA	NA	NA	NA	0
62	C00639	Prostaglandin F2a	HMDB01139	5283078	C00639	1
63	C00407	L-Isoleucine	HMDB00172	6306	C00407	1
64	C00451	D-threo-Isocitric acid	HMDB01874	5318532	C00451	1
65	C00294	Inosine	HMDB00195	6021	C00294	1
66	C02043	Indolelactic acid	HMDB00671	92904	C02043	1
67	C00954	Indoleacetic acid	HMDB00197	802	C00954	1
68	C00262	Hypoxanthine	HMDB00157	790	C00262	1
69	C00192	Hydroxylamine	HMDB03338	787	C00192	1
70	C00135	L-Histidine	HMDB00177	6274	C00135	1
71	C01586	NA	NA	NA	NA	0
72	C00392	Mannitol	HMDB00765	6251	C00392	1
73	C00387	Guanosine	HMDB00133	6802	C00387	1
74	C00160	Glycolic acid	HMDB00115	757	C00160	1
75	C00037	Glycine	HMDB00123	750	C00037	1

76	C03189	NA	NA	NA	NA	0
77	C05401	Galactosylglycerol	HMDB06790	656504	C05401	1
78	C00116	Glycerol	HMDB00131	753	C00116	1
79	C00258	Glyceric acid	HMDB00139	439194	C00258	1
80	C00489	Glutaric acid	HMDB00661	743	C00489	1
81	C00064	L-Glutamine	HMDB00641	5961	C00064	1
82	C00025	NA	NA	NA	NA	0
83	C00221	Beta-D-Glucose	HMDB00516	64689	C00221	1
84	C00800	Gulonic acid	HMDB03290	152304	C00800	1
85	C00122	Fumaric acid	HMDB00134	444972	C00122	1
86	C02095	Beta-D-Fucose	HMDB03081	439650	C02095	1
87	C02336	NA	NA	NA	NA	0
88	C00189	Ethanolamine	HMDB00149	700	C00189	1
89	C00503	Erythritol	HMDB02994	222285	C00503	1
90	C02277	Dodecanol	HMDB11626	8193	C02277	1
91	C12078	NA	NA	NA	NA	0
92	C01420	NA	NA	NA	NA	0
93	C00097	L-Cysteine	HMDB00574	5862	C00097	1
94	C00791	Creatinine	HMDB00562	588	C00791	1
95	C00327	Citrulline	HMDB00904	9750	C00327	1
96	C00158	Citric acid	HMDB00094	311	C00158	1
97	C00187	Cholesterol	HMDB00067	11025495	C00187	1
98	C01571	Capric acid	HMDB00511	2969	C01571	1
99	C03046	(S,S)-Butane-2,3-diol	METPA0353		C03046	1
100	C01753	Beta-Sitosterol	HMDB00852	222284	C01753	1
101	C08240	NA	NA	NA	NA	0
102	C00099	Beta-Alanine	HMDB00056	239	C00099	1
103	C00180	Benzoic acid	HMDB01870	243	C00180	1
104	C00049	L-Aspartic acid	HMDB00191	5960	C00049	1
105	C00152	L-Asparagine	HMDB00168	6267	C00152	1
106	C00219	Arachidonic acid	HMDB01043	444899	C00219	1
107	C06425	Arachidic acid	HMDB02212	10467	C06425	1
108	C01904	D-Arabitol	HMDB00568	827	C01904	1
109	C00216	NA	NA	NA	NA	0
110	C00872	Aminomalonic acid	HMDB01147	100714	C00872	1
111	C00041	L-Alanine	HMDB00161	5950	C00041	1
112	C06104	Adipic acid	HMDB00448	196	C06104	1
113	C00020	Adenosine monophosphate	HMDB00045	6083	C00020	1
114	C00212	Adenosine	HMDB00050	60961	C00212	1
115	C00417	cis-Aconitic acid	HMDB00072	643757	C00417	1
116	C07113	Acetophenone	HMDB33910	7410	C07113	1
117	C08352	NA	NA	NA	NA	0
118	C05659	5-Methoxytryptamine	HMDB04095	1833	C05659	1
119	C01089	NA	NA	NA	NA	0
120	C06474	NA	NA	NA	NA	0
121	C02630	2-Hydroxyglutarate	HMDB59655	43	C02630	1
122	C05984	2-Hydroxybutyric acid	HMDB00008	11266	C05984	1
123	D01947	NA	NA	NA	NA	0
124	C07326	1,5-Anhydrosorbitol	HMDB02712	64960	C07326	1
125	C06105	1,2-Cyclohexanedione	HMDB31344		C06105	1
126		NA	NA	NA	NA	0

4. Pathway Analysis

In this step, users are asked to select a pathway library, as well as specify the algorithms for pathway enrichment analysis and pathway topology analysis.

4.1. Pathway Library

There are 15 pathway libraries currently supported, with a total of 1173 pathways :

- Homo sapiens (human) [80]
- Mus musculus (mouse) [82]
- Rattus norvegicus (rat) [81]
- Bos taurus (cow) [81]
- Danio rerio (zebrafish) [81]
- Drosophila melanogaster (fruit fly) [79]
- Caenorhabditis elegans (nematode) [78]
- Saccharomyces cerevisiae (yeast) [65]
- Oryza sativa japonica (Japanese rice) [83]
- Arabidopsis thaliana (thale cress) [87]

- Escherichia coli K-12 MG1655 [87]
- Bacillus subtilis [80]
- Pseudomonas putida KT2440 [89]
- Staphylococcus aureus N315 (MRSA/VSSA) [73]
- Thermotoga maritima [57]

Your selected pathway library code is mmu (KEGG organisms abbreviation).

4.2. Pathway Enrichment Analysis

Pathway enrichment analysis usually refers to quantitative enrichment analysis directly using the com- pound concentration values, as compared to compound lists used by over-representation analysis. As a result, it is more sensitive and has the potentail to identify **subtle but consistent** changes among compounds involved in the same biological pathway.

Many procedures have been developed in the last decade for quantitative enrichment analysis, the most famous being the Gene Set Enrichment Analysis. Many new and improved methods have been implemented ever since. The enrichment analysis is based on GlobalTest and GlobalAncova. Both methods support enrichment analysis with binary, multi-group, as well as continuous phenotypes. The p values can be approximated based on the asymptotic distribution without using permutations which is computationally very intensive and is not suitable for web applications. Please note, when sample sizes are small, the approximated p values may be slightly less accurate compared to p values obtained by permutation-based method (for details, please refer to the paper by Goeman, J.J. et al. [1] and Hummel, M. et al.[2]. However, since our focus is to identify the most relevant pathways within the pathways in the library, we are more interested in the rank of the pathway, not its absolute p-value. Therefore, this disadvantage may be tolerated.

The selected pathway enrichment analysis method is Globaltest.

4.3. Pathway Topology Analysis

The structure of biological pathways represent our knowledge about the complex relationships among molecules within a cell or a living organism. However, most pathway analysis algorithms fail to take the structural information into consideration when estimating which pathways are significantly changed under conditions of study It is well-known that changes in more important positions of a network will trigger a more severe impact on the pathway than changes occured in marginal or relatively isolated positions.

The pathway toplogy analysis uses two well-established node centrality measures to estimate node importance—degree centrality and betweenness centrality. Degree centrality is defined as the number of links occured upon a node. For directed graph, there are two types of degree: in-degree for links come from other nodes, and out-degree for links initiated from the current node. Metabolic networks are directed graph. Here we only consider the out-degree for node importance measure. It is assumed that nodes in upstream will have regulatory roles for the downstream nodes, not vice versa. The betweenness centrality measures number of shortest paths going through the node. Since metabolic network is directed, we use relative betweenness centrality for metabolite importance measure. The degree centrality measures focus more on local connectivities, while the betweenness centrality measures focus more on global network topology. For more detailed discussions on various graph-based methods for analysing biological networks, please refer to the article by Tero Aittokallio, T. et al.[3]

Please note, for comparison among different pathways, the node importance values calculated from centrality measures are further normalized by the sum of the importance of the pathway. Therefore, the total/maximum importance of each pathway is 1; the importance measure of each metabolite node is actually the percentage w.r.t the total pathway importance, and the pathway impact value is the cumulative percentage from the matched metabolite nodes.

Your selected node importance measure for topological analysis is relative betweenness centrality.

5. Pathway Analysis Result

The results from pathway analysis are presented graphically as well as in a detailed table.

A Google-map style interactive visualization system was implemented to facilitate data exploration. The graphical output contains three levels of view: metabolome view, pathway view, and com- pound view. Only the metabolome view is shown below. Pathway views and compound views are generated dynamically based on your interactions with the visualization system. They are available in your downloaded files.

---

[1] Goeman J.J. and Bühlman P. Analyzing gene expression data in terms of gene sets: methodological issues, Bioinformatics 2007 23(8):980–987.

[2] Manuela Hummel, Reinhard Meister and Ulrich Mansmann. GlobalANCOVA: exploration and assessment of gene group effects, Bioinformatics 2008 24(1):78-85

[3] Tero Aittokallio and Benno Schwikowski. Graph-based methods for analysing networks in cell biology, Briefings in Bioinformatics 2006 7(3):243-255

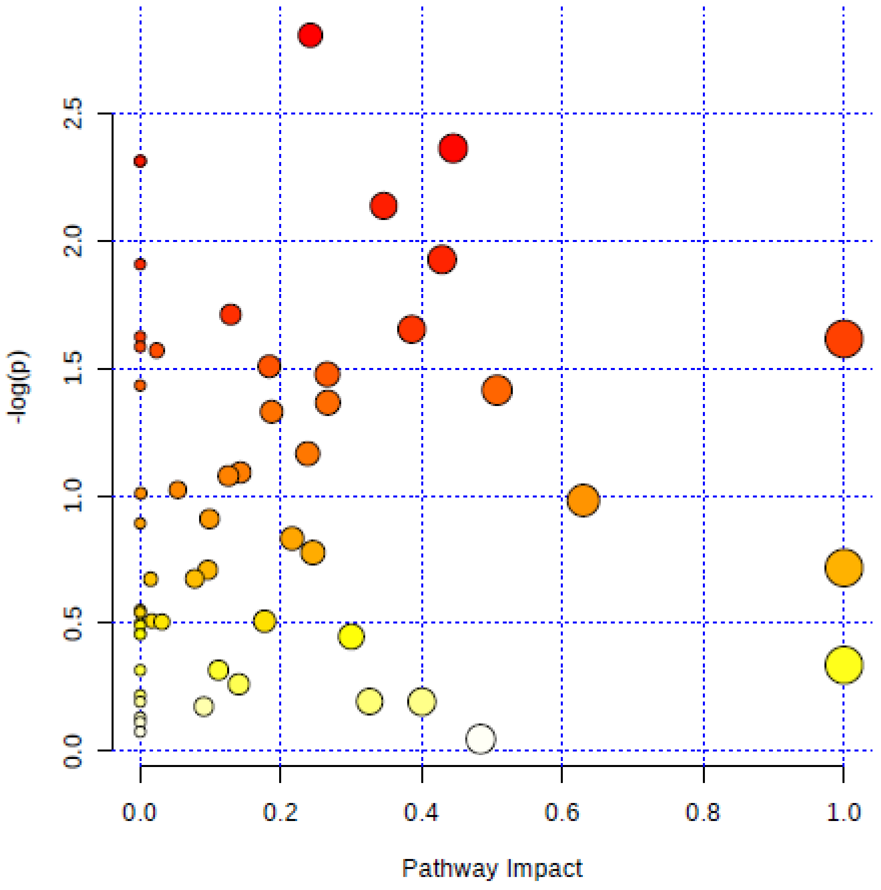

Figure S1. Summary of Pathway Analysis 30 min.

The table below shows the detailed results from the pathway analysis. Since we are testing many pathways at the same time, the statistical p values from enrichment analysis are further adjusted for multiple testings. In particular, the Total is the total number of

compounds in the pathway; the Hits is the actually matched number from the user uploaded data; the Raw *p* is the original *p* value calculated from the enrichment analysis; the Holm *p* is the *p* value adjusted by Holm-Bonferroni method; the FDR *p* is the *p* value adjusted using False Discovery Rate; the Impact is the pathway impact value calculated from pathway topology analysis.

Table S4. Result from Pathway Analysis.

	Total Cmpd	Hits	Raw p	-log(p)	Holm adjust	FDR	Impact
Histidine metabolism	15	2	6.05E-02	2.81E+00	1.00E+00	7.67E-01	0.24
beta-Alanine metabolism	17	3	9.43E-02	2.36E+00	1.00E+00	7.67E-01	0.44
Lysine biosynthesis	4	1	9.92E-02	2.31E+00	1.00E+00	7.67E-01	0.00
Lysine degradation	23	1	9.92E-02	2.31E+00	1.00E+00	7.67E-01	0.00
Biotin metabolism	5	1	9.92E-02	2.31E+00	1.00E+00	7.67E-01	0.00
Alanine, aspartate and glutamate metabolism	24	7	1.18E-01	2.14E+00	1.00E+00	7.67E-01	0.35
Taurine and hypotaurine metabolism	8	2	1.46E-01	1.93E+00	1.00E+00	7.67E-01	0.43
Thiamine metabolism	7	1	1.49E-01	1.91E+00	1.00E+00	7.67E-01	0.00
Aminoacyl-tRNA biosynthesis	69	17	1.81E-01	1.71E+00	1.00E+00	7.67E-01	0.13
Glycerolipid metabolism	18	2	1.92E-01	1.65E+00	1.00E+00	7.67E-01	0.39
Butanoate metabolism	22	3	1.97E-01	1.62E+00	1.00E+00	7.67E-01	0.00
Valine, leucine and isoleucine biosynthesis	11	4	1.99E-01	1.61E+00	1.00E+00	7.67E-01	1.00
Valine, leucine and isoleucine degradation	38	3	2.05E-01	1.58E+00	1.00E+00	7.67E-01	0.00
Glutathione metabolism	26	4	2.08E-01	1.57E+00	1.00E+00	7.67E-01	0.02
Pyruvate metabolism	23	1	2.21E-01	1.51E+00	1.00E+00	7.67E-01	0.18
Arginine and proline metabolism	44	8	2.28E-01	1.48E+00	1.00E+00	7.67E-01	0.27
Pantothenate and CoA biosynthesis	15	4	2.39E-01	1.43E+00	1.00E+00	7.67E-01	0.00
Glycine, serine and threonine metabolism	31	6	2.43E-01	1.41E+00	1.00E+00	7.67E-01	0.51
Pentose and glucuronate interconversions	16	5	2.55E-01	1.37E+00	1.00E+00	7.67E-01	0.27
Cysteine and methionine metabolism	27	3	2.64E-01	1.33E+00	1.00E+00	7.67E-01	0.19
Nicotinate and nicotinamide metabolism	13	1	3.12E-01	1.16E+00	1.00E+00	8.23E-01	0.24
Pyrimidine metabolism	41	6	3.36E-01	1.09E+00	1.00E+00	8.23E-01	0.14
Purine metabolism	68	10	3.41E-01	1.08E+00	1.00E+00	8.23E-01	0.13
Steroid biosynthesis	35	2	3.60E-01	1.02E+00	1.00E+00	8.23E-01	0.05
Glycerophospholipid metabolism	30	2	3.65E-01	1.01E+00	1.00E+00	8.23E-01	0.00
Phenylalanine metabolism	11	3	3.75E-01	9.81E-01	1.00E+00	8.23E-01	0.63
Glycolysis or Gluconeogenesis	26	2	4.03E-01	9.09E-01	1.00E+00	8.23E-01	0.10
Propanoate metabolism	20	3	4.10E-01	8.91E-01	1.00E+00	8.23E-01	0.00
Citrate cycle (TCA cycle)	20	5	4.35E-01	8.32E-01	1.00E+00	8.23E-01	0.22
Retinol metabolism	16	1	4.60E-01	7.77E-01	1.00E+00	8.23E-01	0.25
Phenylalanine, tyrosine and tryptophan biosynthesis	4	2	4.88E-01	7.17E-01	1.00E+00	8.23E-01	1.00
Primary bile acid biosynthesis	46	3	4.92E-01	7.09E-01	1.00E+00	8.23E-01	0.10
Galactose metabolism	26	6	5.09E-01	6.75E-01	1.00E+00	8.23E-01	0.08
Sphingolipid metabolism	21	2	5.10E-01	6.73E-01	1.00E+00	8.23E-01	0.02
Pentose phosphate pathway	19	1	5.76E-01	5.52E-01	1.00E+00	8.23E-01	0.00
Fatty acid elongation in mitochondria	27	1	5.81E-01	5.43E-01	1.00E+00	8.23E-01	0.00
Fatty acid metabolism	39	1	5.81E-01	5.43E-01	1.00E+00	8.23E-01	0.00
Steroid hormone biosynthesis	72	1	6.01E-01	5.09E-01	1.00E+00	8.23E-01	0.02
Tryptophan metabolism	40	2	6.02E-01	5.08E-01	1.00E+00	8.23E-01	0.18
Fructose and mannose metabolism	21	2	6.03E-01	5.05E-01	1.00E+00	8.23E-01	0.03
Selenoamino acid metabolism	15	1	6.06E-01	5.00E-01	1.00E+00	8.23E-01	0.00
D-Glutamine and D-glutamate metabolism	5	1	6.12E-01	4.91E-01	1.00E+00	8.23E-01	0.00
Starch and sucrose metabolism	19	1	6.32E-01	4.59E-01	1.00E+00	8.23E-01	0.00
Fatty acid biosynthesis	43	4	6.33E-01	4.57E-01	1.00E+00	8.23E-01	0.00
Sulfur metabolism	5	1	6.39E-01	4.48E-01	1.00E+00	8.23E-01	0.30
Linoleic acid metabolism	6	1	7.14E-01	3.36E-01	1.00E+00	8.80E-01	1.00
Inositol phosphate metabolism	28	1	7.29E-01	3.17E-01	1.00E+00	8.80E-01	0.11
Ascorbate and aldarate metabolism	9	1	7.29E-01	3.17E-01	1.00E+00	8.80E-01	0.00
Tyrosine metabolism	44	2	7.70E-01	2.61E-01	1.00E+00	9.02E-01	0.14
Nitrogen metabolism	9	3	8.03E-01	2.19E-01	1.00E+00	9.02E-01	0.00
Arachidonic acid metabolism	36	2	8.23E-01	1.94E-01	1.00E+00	9.02E-01	0.33
Methane metabolism	9	2	8.25E-01	1.93E-01	1.00E+00	9.02E-01	0.40
Cyanoamino acid metabolism	6	2	8.25E-01	1.93E-01	1.00E+00	9.02E-01	0.00
Amino sugar and nucleotide sugar metabolism	37	3	8.40E-01	1.74E-01	1.00E+00	9.02E-01	0.09
Biosynthesis of unsaturated fatty acids	42	5	8.76E-01	1.32E-01	1.00E+00	9.24E-01	0.00
Porphyrin and chlorophyll metabolism	27	1	8.94E-01	1.12E-01	1.00E+00	9.26E-01	0.00
Ubiquinone and other terpenoid-quinone biosynthesis	3	1	9.27E-01	7.56E-02	1.00E+00	9.43E-01	0.00
Glyoxylate and dicarboxylate metabolism	18	4	9.55E-01	4.64E-02	1.00E+00	9.55E-01	0.48

The report was generated on Wed Apr 19 05:14:08 2017 with R version 3.3.2 (2016-10-31).

Supplementary File: Analysis report metabolomics 24 hours

User ID: guest8753032602229817641

Pathway Analysis Result 24 hours:

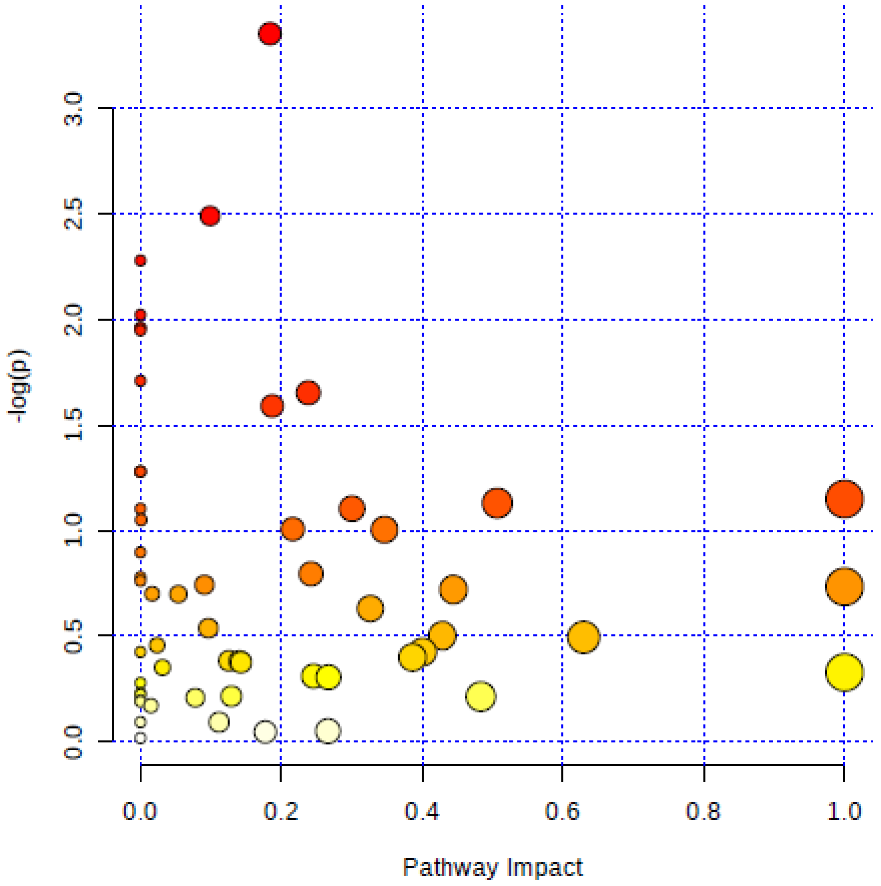

Figure S2. Summary of Pathway Analysis 24 hours

The table below shows the detailed results from the pathway analysis. Since we are testing many pathways at the same time, the statistical *p* values from enrichment analysis are further adjusted for multiple testings. In particular, the Total is the total number of compounds in the pathway; the Hits is the actually matched number from the user uploaded data; the Raw *p* is the original *p* value calculated from the enrichment analysis; the Holm *p* is the *p* value adjusted by Holm-Bonferroni method; the FDR *p* is the *p* value adjusted using False Discovery Rate; the Impact is the pathway impact value calculated from pathway topology analysis.

Table S5. Result from Pathway Analysis 24 hours.

	Total Cmpd	Hits	Raw *p*	-log(*p*)	Holm adjust	FDR	Impact
Pyruvate metabolism	23	1	3.50E-02	3.35E+00	1.00E+00	9.23E-01	0.18
Glycolysis or Gluconeogenesis	26	2	8.29E-02	2.49E+00	1.00E+00	9.23E-01	0.10
Biosynthesis of unsaturated fatty acids	42	5	1.02E-01	2.28E+00	1.00E+00	9.23E-01	0.00
Fatty acid biosynthesis	43	4	1.32E-01	2.02E+00	1.00E+00	9.23E-01	0.00
Fatty acid elongation in mitochondria	27	1	1.41E-01	1.96E+00	1.00E+00	9.23E-01	0.00
Fatty acid metabolism	39	1	1.41E-01	1.96E+00	1.00E+00	9.23E-01	0.00
Butanoate metabolism	22	3	1.42E-01	1.95E+00	1.00E+00	9.23E-01	0.00
Starch and sucrose metabolism	19	1	1.81E-01	1.71E+00	1.00E+00	9.23E-01	0.00
Nicotinate and nicotinamide metabolism	13	1	1.91E-01	1.65E+00	1.00E+00	9.23E-01	0.24
Cysteine and methionine metabolism	27	3	2.04E-01	1.59E+00	1.00E+00	9.23E-01	0.19
Lysine biosynthesis	4	1	2.79E-01	1.28E+00	1.00E+00	9.23E-01	0.00
Lysine degradation	23	1	2.79E-01	1.28E+00	1.00E+00	9.23E-01	0.00

Biotin metabolism	5	1	2.79E-01	1.28E+00	1.00E+00	9.23E-01	0.00
Valine, leucine and isoleucine biosynthesis	11	4	3.18E-01	1.15E+00	1.00E+00	9.23E-01	1.00
Glycine, serine and threonine metabolism	31	6	3.24E-01	1.13E+00	1.00E+00	9.23E-01	0.51
Sulfur metabolism	5	1	3.32E-01	1.10E+00	1.00E+00	9.23E-01	0.30
Propanoate metabolism	20	3	3.33E-01	1.10E+00	1.00E+00	9.23E-01	0.00
Glycerophospholipid metabolism	30	2	3.50E-01	1.05E+00	1.00E+00	9.23E-01	0.00
Citrate cycle (TCA cycle)	20	5	3.66E-01	1.00E+00	1.00E+00	9.23E-01	0.22
Alanine, aspartate and glutamate metabolism	24	7	3.67E-01	1.00E+00	1.00E+00	9.23E-01	0.35
Ubiquinone and other terpenoid-quinone biosynthesis	3	1	4.09E-01	8.95E-01	1.00E+00	9.23E-01	0.00
Histidine metabolism	15	2	4.52E-01	7.93E-01	1.00E+00	9.23E-01	0.24
Pentose phosphate pathway	19	1	4.59E-01	7.78E-01	1.00E+00	9.23E-01	0.00
Porphyrin and chlorophyll metabolism	27	1	4.68E-01	7.60E-01	1.00E+00	9.23E-01	0.00
Amino sugar and nucleotide sugar metabolism	37	3	4.76E-01	7.42E-01	1.00E+00	9.23E-01	0.09
Phenylalanine, tyrosine and tryptophan biosynthesis	4	2	4.81E-01	7.33E-01	1.00E+00	9.23E-01	1.00
beta-Alanine metabolism	17	3	4.87E-01	7.19E-01	1.00E+00	9.23E-01	0.44
Steroid hormone biosynthesis	72	1	4.97E-01	6.99E-01	1.00E+00	9.23E-01	0.02
Steroid biosynthesis	35	2	4.98E-01	6.97E-01	1.00E+00	9.23E-01	0.05
Arachidonic acid metabolism	36	2	5.33E-01	6.29E-01	1.00E+00	9.23E-01	0.33
Primary bile acid biosynthesis	46	3	5.85E-01	5.36E-01	1.00E+00	9.23E-01	0.10
Taurine and hypotaurine metabolism	8	2	6.06E-01	5.01E-01	1.00E+00	9.23E-01	0.43
Phenylalanine metabolism	11	3	6.10E-01	4.93E-01	1.00E+00	9.23E-01	0.63
Glutathione metabolism	26	4	6.34E-01	4.56E-01	1.00E+00	9.23E-01	0.02
Methane metabolism	9	2	6.54E-01	4.24E-01	1.00E+00	9.23E-01	0.40
Cyanoamino acid metabolism	6	2	6.54E-01	4.24E-01	1.00E+00	9.23E-01	0.00
Glycerolipid metabolism	18	2	6.72E-01	3.98E-01	1.00E+00	9.23E-01	0.39
Purine metabolism	68	10	6.82E-01	3.82E-01	1.00E+00	9.23E-01	0.13
Tyrosine metabolism	44	2	6.84E-01	3.80E-01	1.00E+00	9.23E-01	0.14
Pyrimidine metabolism	41	6	6.87E-01	3.75E-01	1.00E+00	9.23E-01	0.14
Fructose and mannose metabolism	21	2	7.04E-01	3.51E-01	1.00E+00	9.23E-01	0.03
Linoleic acid metabolism	6	1	7.21E-01	3.27E-01	1.00E+00	9.23E-01	1.00
Retinol metabolism	16	1	7.34E-01	3.10E-01	1.00E+00	9.23E-01	0.25
Pentose and glucuronate interconversions	16	5	7.37E-01	3.05E-01	1.00E+00	9.23E-01	0.27
Nitrogen metabolism	9	3	7.57E-01	2.78E-01	1.00E+00	9.23E-01	0.00
Selenoamino acid metabolism	15	1	7.96E-01	2.28E-01	1.00E+00	9.23E-01	0.00
Thiamine metabolism	7	1	8.00E-01	2.24E-01	1.00E+00	9.23E-01	0.00
Aminoacyl-tRNA biosynthesis	69	17	8.07E-01	2.15E-01	1.00E+00	9.23E-01	0.13
Glyoxylate and dicarboxylate metabolism	18	4	8.08E-01	2.13E-01	1.00E+00	9.23E-01	0.48
Galactose metabolism	26	6	8.12E-01	2.08E-01	1.00E+00	9.23E-01	0.08
Pantothenate and CoA biosynthesis	15	4	8.23E-01	1.95E-01	1.00E+00	9.23E-01	0.00
Valine, leucine and isoleucine tion	38	3	8.27E-01	1.90E-01	1.00E+00	9.23E-01	0.00
Sphingolipid metabolism	21	2	8.44E-01	1.70E-01	1.00E+00	9.23E-01	0.02
Inositol phosphate metabolism	28	1	9.12E-01	9.21E-02	1.00E+00	9.62E-01	0.11
Ascorbate and aldarate metabolism	9	1	9.12E-01	9.21E-02	1.00E+00	9.62E-01	0.00
Arginine and proline metabolism	44	8	9.51E-01	5.00E-02	1.00E+00	9.72E-01	0.27
Tryptophan metabolism	40	2	9.55E-01	4.58E-02	1.00E+00	9.72E-01	0.18
D-Glutamine and D-glutamate metabolism	5	1	9.83E-01	1.69E-02	1.00E+00	9.83E-01	0.00

The report was generated on Wed Apr 19 04:52:56 2017 with R version 3.3.2 (2016-10-31).
